# A Conflict of Opinion

**Published:** 1917-05-19

**Authors:** 


### The Hospital

The Workers' Newspaper of Administrative Medicine and Institutional Life, Administration, National Insurance and Health.

No. 1615, Vol. LXII. SATURDAY, MAY 19, 1917. PRICE ONE PENNY

### A CONFLICT OF OPINION.

In spite of the routine use of anti-tetanic serum for all wounded soldiers as soon as possible after their wounds are inflicted, tetanus is not wholly prevented amongst our casualties, and continues to claim victims who, if very, very few in comparison with the other causes of mortality in the field, are yet not a negligible number. Nothing seems more clearly and definitely established as a result of war experience than that it is the preventive dose of anti-tetanic serum that keeps the proportion of cases down to a minute fraction of 1 per cent. This conclusion seems to be established so firmly that everyone with knowledge of the facts agrees with it. On the other hand, there is very much less agreement concerning the exact amount of benefit to be derived from the curative use of tetanus anti-toxin—that is, in cases where the disease has actually developed; and not only about the value of the serum treatment itself, but about the best methods of administering it.

Some months ago Sir David Bruce and the Tetanus Committee of which he is the head issued a pronouncement for the guidance of medical officers who may have to treat cases of tetanus, in which they recommend first and foremost the immediate use of tetanus anti-toxin by the intrathecal route, as being the surest and safest. The intravenous method of introduction was also thought to be of value, second only to the intrathecal route. As a result of this authoritative guidance, the common practice amongst military medical officers when confronted with a case of tetanus has been to use the method indicated, with or without in addition the intravenous, intramuscular, and subcutaneous methods.

Early this year a paper by Colonel Sir William B. Leishman and Major A. B. Smallman appeared* in which these two observers analysed a series of cases treated in France, whereas Sir D. Bruce's material has been gathered mainly from hospitals in England. Leishman and Smallman fell seriously foul of Bruce and the Tetanus Committee: they asserted that intrathecal medication was inferior to the intramuscular injection of the serum; and they gave second place, next to the latter, to the subcutaneous method. That is to say, they were diametrically opposed to the practice which, on the authority of a committee of experts, medical officers in charge of military beds had universally adopted.

* The *Lancet*, January 27, 1917.

The matter was so important that it could scarcely be left where it stood. The upshot is seen in a recent issue of the *Lancet*,* the clinical section of which is entirely given up to a series of original papers dealing with the serum treatment of tetanus in wounded soldiers. Sir David Bruce brings forward a defence of his own position, together with some strong criticism of Leishman and Smallman's statistics and conclusions. Other authors, all of them experts having special facilities for studying the subject and for analysing considerable series of cases, also give their views; and a medical statistician, Captain M. Greenwood, contributes a paper in which he suggests that both sides of the disputants have read into their figures inferences and arguments which those figures are not logically capable of yielding.

The clinical contributors to this symposium support Sir David Bruce, for the most part: there seems to be, so far, but little inclination to adopt the opposing view. The subject is, admittedly, both abstruse in itself and most exceptionally difficult of analysis, because almost every clinical case of tetanus receives treatment by more than one (and often by all four) of the alternative routes of administration. Quite probably one result of Sir William Leishman's intervention may be to render medical officers more than ever prone to give their anti-toxin by every available channel—a procedure which would be of doubtful benefit to the patients and most confusing to the researchers. Sir David Bruce fears that another result will be to discourage the use of the intrathecal route because it is more trouble than the other methods.

Colonel Sir William Leishman is, we believe, the official Adviser in Pathology to the British Expeditionary Force: this somewhat anomalous office appears to carry undefined functions and a very wide range of authority. Since this is

* May 5, 1917.

purely a question of clinical medicine, in which Sir William is not and does not profess to be an expert, there will be, we think, a tendency in the profession to rely more upon the Tetanus Committee than on the opinions of a pathologist on this matter. It cannot be suggested that Sir William Leishman's opinion on a point of purely clinical medicine is of little value; but at least it is certain that all his predilections lie in the region of bacteriology and pathology. In these circumstances it was, to say the least of it, very daring of him to break a lance outside his own special province with a body of experts who have had much better opportunities than he has for forming correct opinions about the treatment of tetanus. No final judgment about the point at issue can yet be pronounced; but at present the general trend of opinion is distinctly in favour of the intrathecal route first and foremost. Happily, there seems to be general agreement that serum treatment does save a percentage of these cases that would otherwise end fatally; but the mortality rate is still terribly high, round about 50 per cent. of the cases treated.

